# Teachers and School-Houses

**Published:** 1859-10

**Authors:** 


### TEACHERS AND SCHOOL-HOUSES.

Life's experiences are our teachers—the places where we learn our lessons are numberless.

"In a dungeon dark, damp, and filthy beyond expression," without an atom of food or drink for more than forty hours, the wretched prisoners laid, strangers in a foreign land. A leg of mutton was then thrown to them to be striven and scrambled for, and then torn by tooth and nail, as best might be. For six nights they were compelled to sleep on the cold floor, with nothing over them, to protect them from the death damps and noisome fevers of the horrid place.

One of these prisoners was John Howard, of immortal fame, then but thirty years old. The scenes which he had witnessed made such a deep impression on his mind, that he began to multiply his own sufferings by those of his fellow-prisoners, and these, by all the dungeons of the country, and these again by all the dungeons of all countries, of all lands, and these sent up such a wail of woe, that he at length resolved to devote his life and fortune to the alleviation of human suffering.

Thus it is that trouble has its uses; and there is no sorrow out of which may not come a joy, and solid rocks be made to yield the crystal water.

